# Myocyte Culture with Decellularized Skeletal Muscle Sheet with Observable Interaction with the Extracellular Matrix

**DOI:** 10.3390/bioengineering9070309

**Published:** 2022-07-12

**Authors:** Satoshi Nakada, Yuri Yamashita, Seiya Akiba, Takeru Shima, Eri Arikawa-Hirasawa

**Affiliations:** 1 Japanese Center for Research on Women in Sport, Juntendo University Graduate School of Health and Sports Science, Chiba 270-1695, Japan; s-nakada@juntendo.ac.jp; 2 Research Institute for Diseases of Old Age, Juntendo University Graduate School of Medicine, Tokyo 113-8421, Japan; yuriyama@juntendo.ac.jp (Y.Y.); teitokousokudokoutuueidan05kei@gmail.com (S.A.); ta-shima@gunma-u.ac.jp (T.S.); 3 Aging Biology in Health and Disease, Juntendo University Graduate School of Medicine, Tokyo 113-8421, Japan; 4 Department of Neurology, Juntendo University Graduate School of Medicine, Tokyo 113-8421, Japan; 5 Faculty of Education, Gunma University, Maebashi 371-8510, Japan

**Keywords:** skeletal muscle, cell culture, myoblasts, extracellular matrix, decellularization, microdevice

## Abstract

In skeletal muscles, muscle fibers are highly organized and bundled within the basement membrane. Several microfabricated substrate models have failed to mimic the macrostructure of native muscle, including various extracellular matrix (ECM) proteins. Therefore, we developed and evaluated a system using decellularized muscle tissue and mouse myoblasts C2C12 to analyze the interaction between native ECM and myocytes. Chicken skeletal muscle was sliced into sheets and decellularized to prepare decellularized skeletal muscle sheets (DSMS). C2C12 was then seeded and differentiated on DSMS. Immunostaining for ECM molecules was performed to examine the relationship between myoblast adhesion status, myotube orientation, and collagen IV orientation. Myotube survival in long-term culture was confirmed by calcein staining. C2C12 myoblasts adhered to scaffolds in DSMS and developed adhesion plaques and filopodia. Furthermore, C2C12 myotubes showed orientation along the ECM orientation within DSMS. Compared to plastic dishes, detachment was less likely to occur on DSMS, and long-term incubation was possible. This culture technique reproduces a cell culture environment reflecting the properties of living skeletal muscle, thereby allowing studies on the interaction between the ECM and myocytes.

## 1. Introduction

Tissues in living organisms have an orderly cellular arrangement owing to the basement membrane made from the extracellular matrix (ECM) [1,2,3]. In skeletal muscles, muscle fibers are bundled within basement membranes (endomysium and perimysium) to form highly organized bundles [4,5,6], enabling them to perform mechanical functions. In addition, the muscle basement membrane supports myogenesis and myotube formation [7,8,9] and is also involved in mechanotransduction and mechanical stress tolerance [10,11].

Several models have been reported to control myotube alignment via topological cues using microfabrication techniques to reproduce aligned myofibers in vitro [12,13,14,15,16,17,18,19,20]. Aligned myotubes promote differentiation by arresting the cell cycle and increasing the myotube diameter and length [13,17,19]. These studies suggest that the alignment control of myotubes via topological cues promotes myotube formation. However, the microfabricated substrate is composed of purified ECM protein and cannot ideally mimic the macrostructure containing multiple ECM proteins in skeletal muscles.

Besides the effect of the macrostructure, each ECM molecule in the basement membrane itself has a different effect on the cells: collagen IV (COL IV) and laminin are involved in cell adhesion and migration [7], laminin promotes myogenesis and indirect myoblast fusion [8,9], and chondroitin sulfate inhibits cell differentiation [21,22]. Since each tissue has a tissue-specific ECM composition [23,24,25], the use of a coating substrate composed of native ECM that is extracted from skeletal muscles can mimic the skeletal muscle-specific ECM protein composition accurately [26,27,28,29]. Moreover, the fusion index and myotube area increase when culturing myocytes on the native ECM coating compared to collagen I coating, suggesting that the native ECM promotes myotube differentiation [26,27]. However, the extracted native ECM can lead to the loss of structural characteristics in the basement membrane.

**Figure 1:**
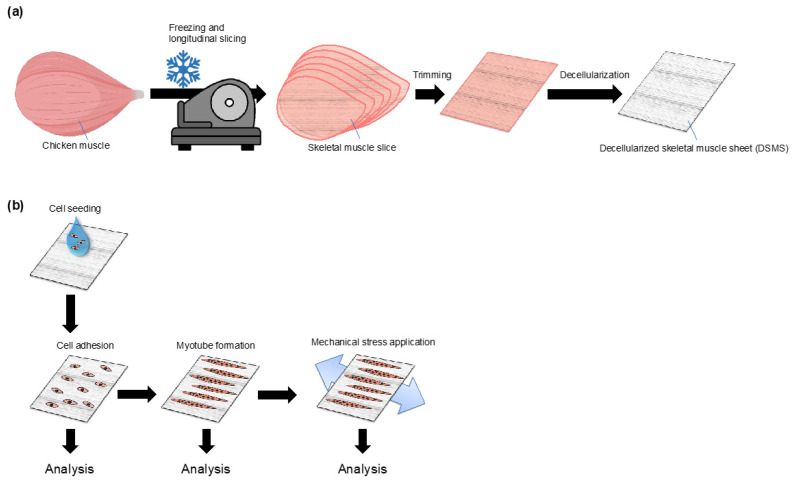
Schematic representation of the DSMS method. (**a**) Fabrication of the decellularized skeletal muscle sheet; (**b**) Application of cell culture using DSMS. DSMS, decellularized skeletal muscle sheets.

To solve this problem, we focused on decellularization technology, by which cellular components can be removed from biological tissues to obtain the ECM constructs [23]. The resulting decellularized products possess the ECM macrostructure, thereby allowing us to examine the effect of basement membrane structures on myocytes [30,31]. Previously, Jank et al. [32] demonstrated a model that highly mimics skeletal muscles by regenerating mouse skeletal muscles via cultured cell inoculation into decellularized mouse skeletal muscles; however, the application of this method is difficult owing to its complexity. Therefore, in this study, we devised a novel myocyte culture method using a sheet-like substrate made from decellularized skeletal muscles (Figure 1a). Our new in vitro model will enable the observation of the interaction between the basement membrane ECM and myocytes and their responsiveness to mechanical stress loading (Figure 1b).

## 2. Materials and Methods

### 2.1. Fabrication of Decellularized Skeletal Muscle Sheets (DSMS)

Sheet-like substrate DSMS were fabricated from decellularized skeletal muscles. The decellularization technique was a modified version of the technique proposed by Urciuolo et al. [33]. Commercially available chicken breast meat was purchased and shaped using a surgical knife, and subsequently frozen at −30 °C overnight. Thereafter, the frozen meat was sliced (1 mm thickness) longitudinally to the orientation of the muscle fibers using an electric meat slicer (RSL-220, Remacom, Mishima, Shizuoka, Japan). The slices were then washed with sterile ultrapure water containing 1% penicillin-streptomycin (P/S, Thermo Fisher Scientific, Waltham, MA, USA) and 250 µg/mL amphotericin B (AmB, Thermo Fisher Scientific) for 1 h.

The solution used in all decellularization processes was four times the volume of the initial weight of the meat slices and was subjected to reciprocal shaking (NR-3, TAITEC Corporation, Saitama, Japan) at 100 revolutions per minute at room temperature (around 25 °C). The slices were then washed with sterile 1% sodium dodecyl sulfate (SDS) solution for 50 h to remove cellular components, during which the 1% SDS solution was changed four times. Next, the remaining SDS in the sheets was removed by washing with sterile 0.01 N NaOH solution for 15 min, followed by washing five times with sterile 50% ethanol for 1 h each. The sheets were washed twice for 10 min with a sterile storage solution (PBS, 1% P/S, and 250 µg/mL AmB). Finally, the sheets were trimmed into 2 cm squares using surgical knives, and DSMS were prepared. The prepared DSMS were stored at 4 °C in a sterile storage solution for short-term storage and at −30 °C in a sterile antifreeze storage solution (PBS, 65% *v*/*v* glycerol, 1% P/S, and 250 µg/mL AmB) for long-term storage. DSMS stored in the antifreeze solution was brought back to room temperature and washed twice with a storage solution for 10 min for further use. 

In order to confirm the progress of decellularization, the time course of change in the amount of protein eluted in the decellularization solution and in the protein component remaining in the skeletal muscle sheets was measured. The amount of protein eluted in the decellularization solution was determined by collecting a portion of the solution 30 min after the decellularization solution was changed. Protein quantification was performed using a protein quantification kit (Pierce BCA Protein Assay Kit, Thermo Fisher Scientific). The amount of eluted protein was calculated per minute. To determine changes in the residual protein components in the skeletal muscle sheets, a portion of the sheets was collected during the decellularization process and was homogenized in SDS sampling buffer (62.5 mM Tris-HCl (pH 6.8), 2% (*w*/*v*) SDS, 10% glycerol, 0.01% (*w*/*v*) bromophenol blue, and 42 mM dithiothreitol). The extracted proteins were subjected to BCA protein quantification, and equal amounts of proteins were subjected to electrophoresis by SDS-PAGE. The gels were then stained with Coomassie Brilliant Blue (CBB) to visualize the protein bands and were imaged using a gel imaging system.

DSMS were attached to the polydimethylsiloxane (PDMS) chamber (Strex, Osaka, Japan) prior to use for DSMS stretching tests and cell culture. DSMS were placed in a PDMS chamber in PBS, with the ECM in the DSMS aligned with the stretching direction of the chamber. Thereafter, PBS was removed and the DSMS were semi-dried for 30 min in a 37 °C humidified CO_2_ incubator. After that, PBS was added to rewet the DSMS. This step allowed the DSMS to stick tightly to the chamber even during cell culture. The characteristic of DSMS to stick to the chamber enabled the same culture operations as that used conventionally.

Stretching tests were performed to confirm the properties of the DSMS. Stretching was performed uniaxially and aligned with the orientation of the DSMS.

Manual stretching was applied to confirm that the DSMS would stretch to the same degree when stretched through the chamber. The chamber with the DSMS was set on a manual stretching tool (STB-100-10, Strex) and stretched at different ratios, and the change in length of the DSMS was measured. Automatic stretching was applied to observe the tolerance of the DSMS to repetitive stretching. The chamber with the DSMS was set in the automatic stretching system (STB-1400-10-R5., Strex) and repeatedly stretched at 1 Hz for 3 h at a 20% stretching ratio. 

### 2.2. Cell Culture

Mouse skeletal myoblasts (C2C12) were purchased from American Type Culture Collection (#CRL-1772, American Type Culture Collection, Rockville, MD, USA). C2C12 were growth cultured on a plastic dish in a growth medium (GM, Dulbecco’s modified Eagle’s medium (DMEM) (10313021, Thermo Fisher Scientific) supplemented with 20% fetal bovine serum (26140-079, Thermo Fisher Scientific) and 1% P/S (Thermo Fisher Scientific)). The growing C2C12 myoblasts were passaged before reaching 80% confluence and used within 10 passages to prevent the loss of myogenic differentiation ability. Differentiation of C2C12 into myotubes was inducted using differentiation medium (DM, DMEM supplemented with 2% donor horse serum (2921149, MP Biomedicals, Tokyo, Japan) and 1% P/S). Cells were cultured in a humidified incubator under 5% CO_2_ at 37 °C, with the medium replaced every other day.

### 2.3. Myoblast Adhesion and Myotube Formation on DSMS or Plastic Dish

The C2C12 cells prepared in plastic plates were detached using 0.25% trypsin-EDTA, suspended in GM, and seeded at 4 × 10^4^ cells/cm^2^ on DSMS attached to the chamber or on plastic dishes. Then, C2C12 was allowed to grow for two days, and differentiation was promoted by replacing the medium with DM.

### 2.4. Fluorescent Microscopic Observation

Fluorescent staining was performed to observe the state of myoblast adhesion and myotube formation. Myocytes on the DSMS were fixed with 4% paraformaldehyde for 25 min, washed three times with PBS for 10 min each, and then permeabilized with 0.3% Triton X-100 in PBS for 10 min. Next, the tissues were washed three times with PBS for 10 min each and were blocked using a blocking solution (1% gelatin in PBS) for 30 min at room temperature. The tissues were then incubated with primary antibody (rabbit anti-COL IV antibody, AB756P, Millipore, Billerica, MA, USA) at 4 °C overnight. They were again incubated with secondary antibody Alexa Fluor 546 Goat Anti-Rabbit IgG (H + L; Thermo Fisher Scientific) for 1 h at room temperature and later at 4 °C overnight with Hoechst 33342 and Alexa Fluor 488 conjugated phalloidin (Thermo Fisher Scientific). For better microscopic observability, myocytes on the DSMS were incubated in a tissue clearing reagent Sca*l*eS4 (40% (*w*/*v*) D-sorbitol, 10% (*w*/*v*) glycerol, 4 M urea, 15% DMSO in H_2_O) for 30 min prior to microscopic observation [34].

Calcein staining was performed to observe myotube survival in long-term culture on the DSMS and plastic dishes. After 6 and 12 days of differentiation, the living myotubes were washed with FluoroBrite DMEM (Thermo Fisher Scientific) and incubated with FluoroBrite DMEM containing 10 µg/mL of calcein-AM (Dojindo Laboratories, Kumamoto, Japan) for 60 min at 37 °C. The cells were then washed with FluoroBrite DMEM and observed under the fluorescence microscope. 

BZ-X700 (KEYENCE, Osaka, Japan) was used for microscopic observation and multifocal images were acquired using the z-stack function. The images were analyzed using ImageJ software (2.3.0/1.53f, National Institutes of Health, Bethesda, MD, USA) [35].

### 2.5. Semi-Quantitative Reverse Transcription Polymerase Chain Reaction

The mRNA expression levels were determined by semi-quantitative reverse transcription (RT)-polymerase chain reaction (PCR). As per the manufacturer’s instructions, total RNA was extracted from myocytes on the DSMS and plastic dishes using QIAzol and RNeasy Mini kit (74104, Qiagen, Hilden, Germany). Possible contamination of genomic DNA was degraded using DNase I (Takara Bio Inc., Shiga, Japan) treatment for 15 min at room temperature. Thereafter, 1000 ng of total RNA was used for reverse transcription primed with Oligo(dT) using an AffinityScript qPCR cDNA Synthesis Kit (600559, Agilent Technologies, Santa Clara, CA, USA). 

Then, RT-PCR of muscle differentiation markers and immature myosin heavy chain (MHC) was performed (EmeraldAmp PCR Kit, Takara Bio Inc.). The primer sequences used in this study are listed in Table 1. The PCR products were electrophoresed using a 2% agarose gel and stained using SYBR Gold (Thermo Fisher Scientific). The gels were then detected with a UV gel imager (Amersham Imager 600, GE Healthcare Bioscience, Piscataway, NJ, USA), and band intensities were quantified using ImageJ densitometry. The quantified mRNA expression levels were corrected for Glyceraldehyde-3-phosphate dehydrogenase (GAPDH) mRNA levels and were normalized to the levels of myocytes on DSMS day 1 of the growth phase.

### 2.6. Statistical Analysis

Statistical analysis was performed using a t-test for independent samples with unequal variance using the Prism 9 software (GraphPad Software, La Jolla, CA, USA).

## 3. Results

### 3.1. Preparation and Properties of DSMS

**Figure 2:**
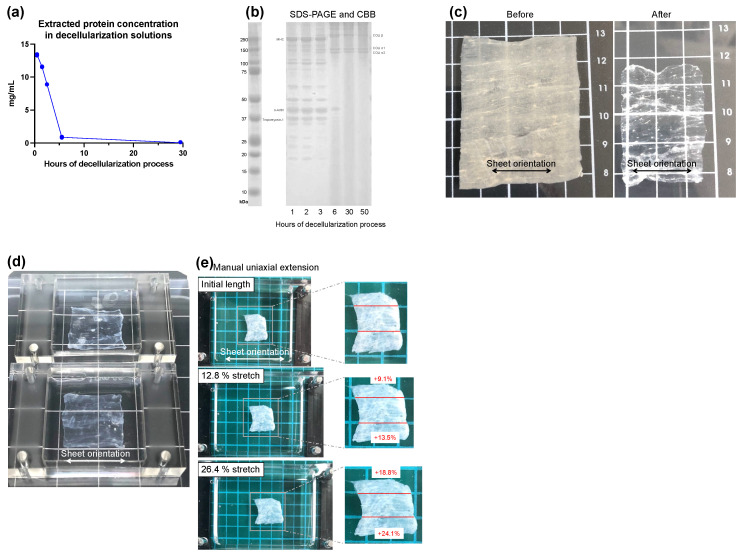
Preparation and properties of the decellularized skeletal muscle sheets. (**a**) Change in the amount of protein eluted by the decellularization solution; (**b**) Change in the remaining protein in the skeletal muscle sheet. The remaining protein was extracted and visualized by SDS-PAGE and CBB staining; (**c**) Appearance of the sheet before and after decellularization. The direction of the extracellular matrix remaining in the sheets can be visually confirmed; (**d**) DSMS attached to the chamber; (**e**) DSMS extension associated with manual stretching. CBB, Coomassie Brilliant Blue; DSMS, decellularized skeletal muscle sheets.

To confirm that decellularization was sufficient, we checked the time course of changes in the amount of protein eluted into the decellularization solution and changes in the protein component in the skeletal muscle sheets. Figure 2a shows the time course of changes in the amount of protein eluted into the decellularized solution during the decellularization process. The amount of protein eluted decreased as the decellularization treatment progressed, with little elution observed between 6 and 30 h. This result indicates that the proteins of the cellular components were completely eluted by the decellularization treatment. Next, the changes in the protein component in skeletal muscle sheets during the decellularization process were checked. In Figure 2b, the DSMS were sampled during the decellularization process, the protein in skeletal muscle sheets was extracted, and the equal amounts of extracted protein was electrophoresed by SDS-PAGE followed by CBB staining. The bands visualized by CBB differed markedly between lanes 1 h, 2 h, and 3 h and lanes 6 h, 30 h, and 50 h of the decellularization process. The bands observed in lanes 1 h, 2 h, and 3 h were considered skeletal muscle contractile proteins such as MHC (220 kDa), α-actin (42 kDa), and tropomyosin-1 (34.7 kDa). On the other hand, lanes 6 h, 30 h, and 50 h were thought to be the ECM proteins COL1α1 (140 kDa), COL1α2 (129 kDa), and COL1β [36], respectively. The results confirm that as the decellularization process progresses, cellular components such as skeletal muscle contractile protein in skeletal muscle sheets were removed, and the ECM proteins remained. These results indicate that 30 h of decellularization was sufficient to remove cellular proteins from the skeletal muscle sheet and fabricate DSMS.

The properties of the DSMS obtained by this determined decellularization process were confirmed. Figure 2c shows the appearance of the skeletal muscle sheets before and after decellularization; the transparency of the sheets increased after decellularization. Furthermore, the horizontal orientation of the ECM was visually confirmed. Next, the stretching properties of the DSMS were confirmed. Figure 2d shows DSMS attached to a PDMS chamber—the direction of the sheet’s ECM aligned with the direction of the chamber’s extension. In Figure 2e, the manual uniaxial stretching was performed, and the extension rate of the chamber was compared with that of the sheet. When the chamber was stretched at 12.8% and 26.4%, the sheets exhibited an average of 15.9% and 21.5% elongation, respectively. The chamber with DSMS was placed in an automatic stretching system and was subjected to 3 h of 1 Hz stretching at 20% elongation. During the application of stretching, the DSMS did not detach from the chamber, indicating that the DSMS have suitable properties for the application of mechanical stress Thus, DSMS have suitable properties for the application of mechanical stress through the PDMS chamber due to its strong adhesion to PDMS and sufficient extensibility.

### 3.2. Morphology of Myoblasts and Myotubes on DSMS

**Figure 3:**
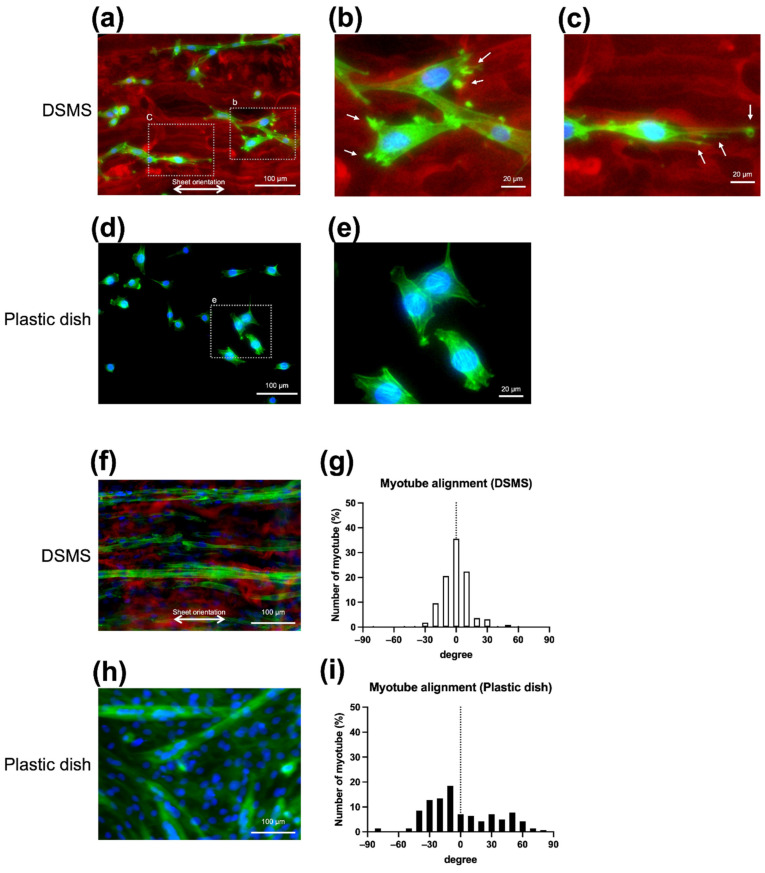
Comparison of myoblasts and morphology of myotubes. Green: phalloidin, red: COL IV, and blue: Hoechst 33342. (**a**) Myoblasts adhered to DSMS. Myoblasts aligned with the remaining COL IV in the DSMS; (**b**) Magnified image. Myoblasts on the DSMS developed actin spikes that appeared to be adhesion plaques (arrow); (**c**) Magnified image. Myoblasts on the DSMS developed lamellipodia and filipodia (arrow); (**d**) Myoblasts adhered to the plastic dish; (**e**) Magnified image. Myoblasts on the plastic dishes did not develop adhesive spots; (**f**) Myotube formation on DSMS. Myotubes formed along the extracellular matrix orientation in DSMS; (**g**) Histogram of angles in myotubes formed on the DSMS. Most myotubes were in the ±20° range; (**h**) Myotube formation on the plastic plate. Myotubes on the plastic plate formed in random orientation; (**i**) Histogram of angles in myotubes formed on the plastic plate. Various angles of myotubes were present on the plastic plate. DSMS, decellularized skeletal muscle sheets.

Morphological observations were made to determine the effects of DSMS on myoblasts and myotubes. To confirm the difference in the adhesion of C2C12 to DSMS and the plastic dish, C2C12 was seeded into DSMS, and myoblast adhesion was compared in a plastic dish the next day. Thereafter, the cells were stained for actin fibers with fluorescence conjugated phalloidin, followed by nuclear staining with Hoechst 33342 and immunostaining with anti-COL IV antibody. On the plastic plate, C2C12 exhibited a star shape and irregular cell orientation (Figure 3d), whereas, on DSMS, the C2C12 cells were spindle-shaped and adhered in line with scaffolds (Figure 3a). The adhesion along the decellularized structures was similar to that reported in previous studies [37]. Through detailed observation of cell adhesion, C2C12 on DSMS was observed to have multiple actin fiber spikes between the scaffolds, which were thought to be adhesion plaques. The adhesive plaques and the shape of the cells suggest that lamellipodia and filipodia were formed (Figure 3b,c) [38,39]. 

In order to confirm the influence of DSMS on myotube formation, myotube differentiation was induced by changing to the DM. On DSMS, myotubes formed in the same direction as the DSMS, and many myotubes formed in the ±20° range, as shown in Figure 3f,g, suggesting that the orientation of the myotubes is controlled by the DSMS. In contrast, on the plastic dish myotubes formed in random directions, as shown in Figure 3h,i. 

### 3.3. Myocyte Differentiation on the DSMS

**Figure 4:**
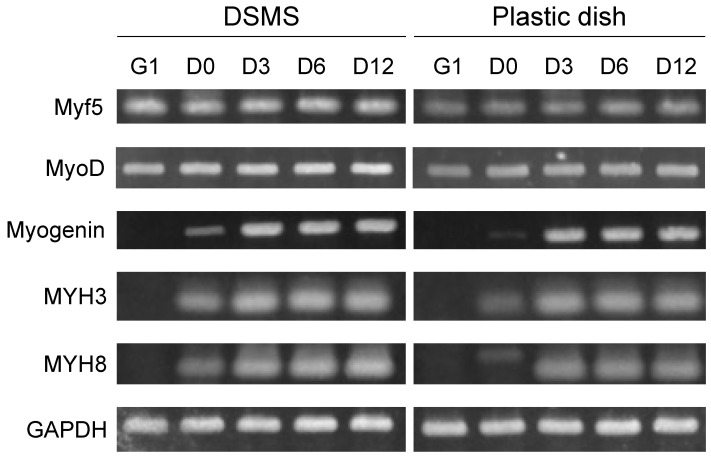
Semi-quantitative reverse transcription-polymerase chain reaction of gene expression involved in myocyte differentiation. G1, Growth day 1. D0–D12, differentiation 0–12 days. DSMS, decellularized skeletal muscle sheets.

RT-PCR was performed using the mRNA extracted from myocytes seeded on DSMS and the plastic dish (Figure 4). The expression of the earliest myogenic regulatory factor (MRF) Myf5 was higher in the DSMS throughout the entire culture period [40,41]. MyoD, an MRF involved in myoblast proliferation and early differentiation, was highly expressed in the DSMS during Growth day 1 (G1) [41,42]. Myogenin, an MRF involved in myotube fusion, was highly expressed in the DSMS during differentiation 0 days (D0) [41,43,44]. The expression of immature MHC, MYH3 and MYH8 was higher in the DSMS at D0 [45,46,47]. These results indicate the enhancement of MRF expression and accelerated expression of immature MHC.

### 3.4. Myotube Viability on the DSMS

**Figure 5:**
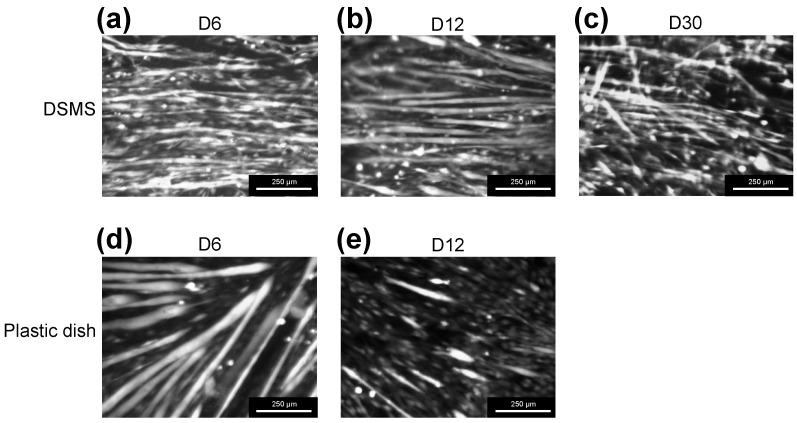
Comparison of cell survival. Live cells were stained with calcein. (**a**–**c**) Live cells on the DSMS; (**d**,**e**) Live cells on the plastic plate; (**a**,**d**) Differentiation day 6; (**b**,**e**) Differentiation day 12; (**c**) Differentiation day 30. In the culture on DSMS, detachment of myotubes was not observed even at 12 days of differentiation. Myotubes were still alive at 30 days of differentiation. In the culture on the plastic dish, myotubes were formed on Day 6 of differentiation, but many myotubes detached on Day 12. DSMS, decellularized skeletal muscle sheets.

In order to observe myotube cell survival on the DSMS and plastic plates during long-term culture, viable cells were stained with calcein. On Day 6 of differentiation, myotubes on both DSMS and plastic dishes were stained with calcein, indicating that myotubes were formed and survived on each substrate (Figure 5a,d). However, on Day 12, the number of myotubes stained with calcein on the plastic dish decreased, suggesting the occurrence of cell detachment (Figure 5e). On the other hand, myotubes on the DSMS survived without detachment even on Day 12 of differentiation (Figure 5b) and were still alive on Day 30 (Figure 5c). 

## 4. Discussion

In this study, longitudinally sliced skeletal muscles were decellularized to obtain flexible substrates suitable for myocyte culture. This allowed us to control the alignment of myoblast adhesion and myotube formation, promote myocyte differentiation, and suppress myotube detachment, enabling a long-term culture of myotubes.

The decellularization process resulted in a sheet-like structure with only the skeletal muscle ECM remaining. As a result of shaping the ECM such that the alignment of skeletal muscle fibers could be seen before decellularization, the alignment of the ECM was maintained after decellularization. The sheets also had strong adhesion to the PDMS chamber, high flexibility, and high durability to withstand prolonged stretching stimuli. This is not only a great advantage in normal cell culture operations but also makes it possible to apply mechanical stretching stimuli through the sheet.

Aligned myofiber is a major characteristic of skeletal muscle. Therefore, many studies have been conducted to align myoblasts and myotubes in order to create in vitro models that mimic skeletal muscles. In this study, we observed the effect of skeletal muscle ECM-derived scaffolds on myoblast morphology, confirming that the myoblasts formed multiple adhesion plaques and elongated filopodia along ECM scaffolds. Since adhesion plaques explore the topology of the scaffold, more adhesion plaques are required on complex surface structures [48]. The multiple adhesion plaques formed by C2C12 myoblasts on DSMS suggest that myoblasts on the DSMS are actively interacting with the complex scaffold of the DSMS. Furthermore, myoblasts on the DSMS were observed to extend filopodia along the DSMS scaffold. During migration, cells form filopodia in the direction of migration and adhere to the scaffold. The cells then translocate toward the scaffold formed by the filopodia by actomyosin-based contraction forces [38,39]. Therefore, C2C12 was considered to be migrating along the orientation of the DSMS scaffold because C2C12 was forming filopodia along the orientation of the DSMS scaffold. Since DSMS affect C2C12 migration, DSMS could be used as a material for studies on myoblast migration.

This study also succeeded in finding that myotubes form along the alignment of the DSMS ECM. On the plastic dish, myotubes were formed in a random orientation, whereas on the DSMS, myotubes were formed in a direction ±20° of the DSMS orientation. A possible mechanism by which myotube orientation is regulated by the DSMS is that the scaffold in DSMS influences the myotube orientation. Cells are influenced by topographical cues [49], and culture systems have been developed to control the myotube orientation by creating microgrooves on the culture plate or through the linear pattern of ECM coating [12,17,18,19,20]. Similarly, DSMS shows a microgroove-like 3D structure along the sheet orientation. Therefore, it is possible that this groove-like structure controlled myotube orientation. 

It is also possible that adhesion and the migration pattern of C2C12 myoblasts on the DSMS affect the myotube orientation. On the plastic dish, C2C12 cells migrate freely on the uniform dish surface and come in contact with the surrounding cells randomly, leading to fusion [50]. As a result, C2C12 on the plastic dish can form myotubes randomly. On the other hand, on DSMS, myoblasts adhere along with the fibrillar collagen, as shown in Figure 3. Since the fibrillar collagen structure in DSMS has a consistent directionality, the migration direction of myoblasts may be restricted. Therefore, it is suggested that myotubes are formed along the DSMS alignment due to myoblast contact and fusion occurring on the DSMS fibrous collagen scaffold.

The ability to control myotube orientation on the DSMS via DSMS orientation is a very advantageous feature for mechanical stimulation; the alignment of the myotube orientation on the DSMS allows mechanical stimulation to be applied at an accurate extension rate along the long myotube axis. This may allow for a more detailed study of the intensity of the mechanical stimulus and the response of the cell.

Several proteins in the ECM of the skeletal muscle basement membrane have affected myogenic differentiation. Laminin promotes myoblast adhesion [7], myogenesis [8], and also indirectly promotes myoblast fusion by increasing cell-to-cell affinity [9]. COL IV promotes myoblast adhesion and migration [7]. In contrast, chondroitin sulfate has been reported to inhibit differentiation [21,22]. Decellularized skeletal muscle is also expected to affect myogenic differentiation because the skeletal muscle basement membrane ECM remains. Previous studies have reported that solubilized decellularized skeletal muscle was used for coatings to promote myogenic differentiation [26,27,28,29]. Since the present study showed accelerated expression of MRF and earlier expression of immature MHC, it is suggested that the culture on the DSMS promoted myogenic differentiation. This suggests that the effect of differentiation-promoting ECMs, such as laminin and COL IV, is stronger than the inhibition of differentiation by chondroitin sulfate.

Detachment of myotubes was frequently observed in long-term cultures and is caused by the disruption of adhesion as myotubes increase contractility with differentiation and maturation [12,51,52,53,54,55,56]. Since this detachment is a major limiting factor in research, detachment suppression methods have been studied [57,58]. In this study, we confirmed that the survival of myotubes on the DSMS revealed that myotube detachment did not occur, and myotubes survived longer on the DSMS than on plastic dishes.

One possible mechanism for the long-term survival of myotubes on the DSMS without detachment is that myotubes may adhere more firmly to the DSMS than to plastic dishes. As mentioned previously, myoblasts formed multiple actin fiber spikes and adhered to the DSMS, suggesting that myoblasts adhere more tightly to the DSMS. Similarly, myotubes may adhere tightly to the DSMS by forming abundant adhesion molecules. In a previous study, the adhesion force of myotubes to substrates was examined [53]. Among the substrates examined, the adhesion force to plastic dishes was the strongest, although the myotubes detached from the plastic dishes. Therefore, the adhesive strength of myotubes alone may not be sufficient to explain the mechanism by which myotubes do not detach and can be cultured in DSMS for long periods of time.

Another possible mechanism for the long-term viability of myotubes on the DSMS is that the low stiffness of the DSMS may prevent the detachment of the myotubes. Previous studies have reported that substrate stiffness affects myotube detachment and survival during long-term culture [52,53]. Bettadapur et al. [52] cultured myotubes on hard PDMS, which has the same stiffness as glass or plastic plates, and on soft PDMS; a previous study reported that soft PDMS suppressed myotube detachment for one to three weeks. The rigid PDMS substrate is unable to absorb the spontaneous contractile force of the myotubes, causing the molecular adhesion to collapse and detach. In contrast, the soft PDMS substrate is thought to absorb the contractile force and inhibit myotube detachment. Skeletal muscle stiffness, expressed as passive elasticity, is about 12 kPa, and decreases slightly after decellularization [53,59]. The DSMS used in this study are also expected to have a similar stiffness because it is a substrate obtained by decellularizing skeletal muscles. Therefore, the low stiffness of DSMS is considered to have inhibited myotube detachment.

Another possibility is that the time to detachment may have been prolonged since the time course of differentiation was extended. Thinner myotubes were observed in the DSMS on Day 6 than on the plastic dish (Figure 5a,d), suggesting that the culture on DSMS may have caused a delay in myotube differentiation. However, based on the semi-quantitative RT-PCR results, no significant difference exists in the expression of differentiation markers between DSMS and the plastic dish, suggesting that the myotube differentiation time course on the DSMS was not delayed. The thinner myotubes on the DSMS may be that myoblasts adhered to the fibrillar collagen scaffold, and migration and cell-cell contact were limited, thereby inhibiting excessive fusion.

These suggest that the main mechanism by which the myotubes survived longer on the DSMS was not due to differences in the time course of differentiation but because of the firm adhesion of myotubes to the DSMS and the low stiffness of the DSMS, which prevented detachment. Using DSMS for myotube culture prevents myotube detachment and enables a more prolonged cell culture. This property of DSMS-based culture could be a great advantage in studies using cultured myotubes. 

## 5. Conclusions

This study reports a novel myocyte culture method using sheet-like ECM structures obtained by decellularization. Myoblasts adhered to the sheet according to the orientation of the ECM, and the direction of the myotube formation could be controlled. Furthermore, by applying mechanical stimulation to the myotubular cells in each sheet, we evaluated the mechanical stress resistance of the myocytes.

## Figures and Tables

**Table 1:** Primer sequence using semi-quantitative reverse transcription-polymerase chain reaction.

Gene	Accession No.	Forward Primer	Reverse Primer
Myf5	NM_008656	5′-TGTATCCCCTCACCAGAGGAT-3′	5′-GGCTGTAATAGTTCTCCACCTGTT-3′
MyoD	NM_010866	5′-AGTGAATGAGGCCTTCGAGA-3′	5′-CTGGGTTCCCTGTTCTGTGT-3′
Myogenin	NM_031189	5′-ACCAGGAGCCCCACTTCTAT-3′	5′-ACGATGGACGTAAGGGAGTG-3′
MHC embryoic (MYH3)	NM_001099635	5′-TCCGACAACGCCTACCAGTT-3′	5′-CCCGGATTCTCCGGTGAT-3′
MHC neonatal (MYH8)	NM_177369	5′-CAGGAGCAGGAATGATGCTCTGAG-3′	5′-AGTTCCTCAAACTTTCAGCAGCCAA-3′
GAPDH	NM_008084	5′-ACTCCACTCACGGCAAATTC-3′	5′-CCTTCCACAATGCCAAAGTT-3′

## Data Availability

Not applicable.
